# On the Action of the Omo-Hyoideus

**Published:** 1848-11

**Authors:** 


*On the Action of the Omo-Hyoideus.*—The reputed action of these muscles, as is shown by reference to the opinions of Bell, Cloquet, Cruvelhier, &c., is, to oppress the os hyoides and larynx. Mr. Skey regards this view as too exclusive, and refers to them a highly important and hitherto unacknowledged function,—viz., depression of the os hyoides towards the *sternum*, the action by which *sucking* is accomplished. In examining such animals as drink by lapping, and in which the formation of a vacuum in the mouth, as in the act of sucking, is not required, Mr. Skey finds the omo-hyoideus absent. Its attachment to the scapula is regarded as a matter of necessity, inasmuch as the clavicle and first rib are occupied by the scalenæ, &c.

To the law that no animal which lives by lapping has this muscle, Mr. Skey acknowledges the objection which arises from the fact that such animals, when young, also live by suction, and this would appear to invalidate his explanation of the action of the muscle. He endeavours to avoid the difficulty, by supposing that the act of sucking in the young animal is effected by the tongue alone.—*Medical Gazette.*

